# Multi-locus sequence analysis of *mycoplasma capricolum *subsp. *capripneumoniae *for the molecular epidemiology of contagious caprine pleuropneumonia

**DOI:** 10.1186/1297-9716-42-86

**Published:** 2011-07-14

**Authors:** Lucía Manso-Silván, Virginie Dupuy, Yuefeng Chu, François Thiaucourt

**Affiliations:** 1 CIRAD, UMR CMAEE, F-34398 Montpellier, France; 2 State Key Laboratory of Veterinary Etiological Biology, Lanzhou Veterinary Research Institute, CAAS, Xujiaping 1, Lanzhou 730046, China

## Abstract

*Mycoplasma capricolum *subsp. *capripneumoniae *(Mccp) is the causative agent of contagious caprine pleuropneumonia (CCPP), a devastating disease of domestic goats. The exact distribution of CCPP is not known but it is present in Africa and the Middle East and represents a significant threat to many disease-free areas including Europe. Furthermore, CCPP has been recently identified in Tajikistan and China. A typing method with an improved resolution based on Multi-Locus Sequence Analysis (MLSA) has been developed to trace new epidemics and to elucidate whether the recently identified cases in continental Asia were due to recent importation of Mccp. The H2 locus, a polymorphic region already in use as a molecular marker for Mccp evolution, was complemented with seven new loci selected according to the analysis of polymorphisms observed among the genome sequences of three Mccp strains. A total of 25 strains, including the two new strains from Asia, were analysed by MLSA resulting in the discrimination of 15 sequence types based on 53 polymorphic positions. A distance tree inferred from the concatenated sequences of the eight selected loci revealed two evolutionary lineages comprising five groups, which showed good correlation with geographic origins. The presence of a distinct Asian cluster strongly indicates that CCPP was not recently imported to continental Asia. It is more likely that the disease has been endemic in the area for a long time, as supported by historical clinical descriptions. In conclusion, this MLSA strategy constitutes a highly discriminative tool for the molecular epidemiology of CCPP.

## Introduction

Contagious Caprine Pleuropneumonia (CCPP) is a devastating disease of goats included in the list of notifiable diseases of the World Organisation for Animal Health (OIE). The first description of the disease dates back to 1873, in Algeria [1]. However, the etiologic agent, *Mycoplasma capricolum *subsp. *capripneumoniae *(Mccp) was only isolated and characterised a century later, in 1976 [2]. This may be explained by several factors. Mccp is one of the most fastidious mycoplasmas to grow in vitro and cultures are often overgrown by concomitant bacteria, hampering its isolation. Mccp may also be difficult to identify because it belongs to the *Mycoplasma mycoides *cluster, a group of five closely related mycoplasmas that are pathogenic for ruminants, also comprising *M. mycoides *subsp. *mycoides *"Small Colony", *M. mycoides *subsp. *capri *(Mmc), *M. capricolum *subsp. *capricolum *(Mcc), and *M. leachii*. These organisms share many genotypic and phenotypic traits, which can lead to problems for identification, particularly when applying classical diagnostic techniques. Furthermore, there has been disagreement over the aetiology of CCPP, which was attributed to Mmc for a long time, generating great confusion. Therefore, much attention must be given to historical clinical descriptions in order to distinguish true CCPP from pleuropneumonia caused by other members of the *M. mycoides *cluster, often associated to other pathologies.

All of this may also explain why the exact distribution of CCPP is not known. What is certain is that the disease is present in Africa and the Middle East, as demonstrated by isolation (which remains the confirmatory test required by the OIE) and molecular characterisation of Mccp strains. Although Mccp was shown to be a rather homogeneous taxon [3-6], two molecular markers revealed some degree of heterogeneity among strains allowing the first studies on the molecular epidemiology of CCPP. Mccp strains showed a high degree of polymorphism between the 16S rDNA genes of their two *rrn *operons, as compared to other members of the *M. mycoides *cluster, which was exploited to analyse the molecular evolution of Mccp [7]. A subsequent study on the molecular epidemiology of CCPP was conducted by this group based on the analysis of the H2 locus, which allowed the discrimination of four groups that showed a good correlation with geographic origins [8]. Since the publication of these studies, very few new strains have been made available, although some of them were of particular interest. First, the isolation of Mccp in the Thrace region of Turkey in 2004 showed that there is a risk of introduction of the disease in Europe [9], and the recent outbreak of CCPP in Mauritius [10] confirmed that Mccp is spreading, threatening many disease-free areas. Furthermore, the presence of CCPP in east Asia has recently been confirmed using molecular techniques in Pakistan [11] and in Tajikistan [12] and several Chinese strains have been characterised as Mccp [13]. In addition, CCPP was recently confirmed in wild ruminants kept in a wildlife reserve in Qatar [14], questioning the long believed strict host specificity of Mccp to the domestic goat. All these recent findings have opened new interesting questions that could not be solved by 16S rDNA or H2 locus analysis due to the limited discriminatory power of these molecular markers. A typing method with an improved resolution is required to address all the epidemiological questions that arise, as well as to trace new epidemics.

The first objective of this study was to develop a discriminative tool for the molecular epidemiology of CCPP. A method based on the analysis of several genetic markers that is known as Multi-Locus Sequence Analysis (MLSA) was chosen for this purpose. The origin of Mccp in east Asia was then investigated using the improved MLSA to determine whether the recently identified Mccp strains were the result of a recent importation or were endemic to the region.

## Materials and methods

### Mccp strains, culture conditions and sample preparation

The 27 strains analysed in this study (Table 1) were characterised as Mccp by specific PCR amplification [15]. Most of them had already been analysed in previous studies [7,8,16] and corresponding 16S rDNA and H2 types are presented whenever available (Table 1). Mycoplasma strains were cultured in modified Hayflick's broth [6] at 37°C, 5% CO_2 _and were harvested in the late exponential phase of growth. DNA was extracted from 3 mL culture or pleural fluid using the DNeasy blood and tissue kit (Qiagen GmbH, Hilden, Germany) and was diluted to a concentration of 1 ng/μL in sterile, deionised water for use as PCR templates.

**Table 1:** List of Mccp strains analysed and corresponding 16S rDNA, H2 locus and MLSA types

Strain^a^	Supplier	Country^b^	Location^b^	Year	Species	16S rDNA	H2	MLSA
97095-Tigray	NVI-E	Ethiopia	Tigray (North)	1997	Goat	IA1a	Aa	1-010
9277-PF1	VRA	Sudan	NK	< 1992	Goat	-	Aa	1-010
99108-P1	SVS	Eritrea	Adi-Keshi, Gash-Barka	1999	Goat	-	Aa	1-010
04012	AWWP	Qatar	Doha, Al Wabra	2004	Wild Goat	-	Aa	1-010
M74/93	NVI-S	Uganda	South East	1993	Sheep	IA	Aa	1-020
M79/93	NVI-S	Uganda	East	1993	Goat	IA	Aa	1-020
8789	LRVZF	Chad	Karal, Dandi	1987	Goat	IB	Ca	2-010
94156	LRVZF	Chad	N'Djamena	1994	Goat	-	Ca	2-010
05021	VRA	Sudan	Darfur, Nyala	< 2005	NK	-	Ca	2-010
95043	LABOCEL	Niger	Goure (East)	1995	Goat	I	Cb	2-020
M1601	LVRI	China	Gansu (Centre)	2007	Goat	-	A	3-010
09018	CIRAD	Tajikistan	Rogun district	2009	Goat	-	D	3-020
C550/1	CVRL	UAE	Dubai	1991	NK	IIA	D	3-030
*Gabes*	*CIRAD*	*Tunisia*	*Gabes (South)*	*1980*	*Goat*	*IIB2*	*B*	*4-010*
*LKD*	*CIRAD*	*Tunisia*	*Kebili, Douz (South)*	*1980*	*Goat*	*IIB2*	*B*	*4-010*
*Gabes/102p*	*CIRAD*	*Tunisia*	*Gabes (South)*	*1980*	*Goat*	*IIB2a (SR)*	*B*	*4-010*
9081-487P	MAF-O	Oman	NK	1990	Goat	II	B	4-010
07033	FU	Turkey	Elazig (East)	2007	Goat	-	B	4-010
7/2	MRI	Oman	NK	1988	Goat	-	B	4-020
97097-Errer	NVI-E	Ethiopia	Errer (East)	1997	Goat	IIB5 (SR)	Ac	5-010
AMRC-C758	AU	Sudan	NK	1981	Goat	IIB	A	5-020
Yatta B	NVI-S	Kenya	Eastern, Yatta	< 1997	NK	IIB4	A	5-020
F38^T^	Type strain	Kenya	NK	1976	Goat	IIB	Ab	5-030
94029-C5	AVS	Oman	NK	1994	Goat	-	A	5-040
91039-C3	NVI-E	Ethiopia	NK	1991	Goat	-	A	5-050
9231-Abomsa	CIRAD	Ethiopia	Godjam (West)	1982	Goat	IIB1	A	5-060
92138-CLP1	NVI-E	Ethiopia	NK	1992	Goat	-	A	5-060

^a^All strains are Mccp isolates with the exception of strain 09018, which corresponds to extracted DNA from a CCPP clinical sample, from which Mccp could not be isolated, and Gabes/102p, which was obtained from strain Gabes after 102 in vitro passages. Strains that were not included in a previous study based on H2 locus typing [8] are underlined. Italicised are related isolates or variants used to analyse the stability of the MLSA markers and regarded as a single strain. Therefore, only 25 strains were considered for molecular epidemiology analysis.

^b^The country and location are those of isolation. However, in two cases, diseased animals were known to be imported from another country: Strain 99108-P1 was isolated in Eritrea from animals coming from Tigray, north Ethiopia, whereas strain 7/2 was isolated in Oman though actually coming from Turkey [27].

Abbreviations: AU: Aarhus University, Denmark; AVS: Agriculture and Veterinary Services, Oman; AWWP: Al Wabra Wildlife Preservation, Qatar; CIRAD, France; CVRL: Central Veterinary Research Laboratory, United Arab Emirates; FU: Firat University, Turkey; LABOCEL: Laboratoire Central de l'Elevage de Niamey, Niger; LRVZF: Laboratoire de Recherches Vétérinaires et Zootechniques de Farcha, Chad; LVRI: Lanzhou Veterinary Research Institute, China; MAF-O: Ministry of Agriculture and Fisheries, Oman; MRI: Moredun Research Institute, UK; NVI-E: National Veterinary Institute, Ethiopia; NVI-S: National Veterinary Institute, Sweden; SVS: Senhit Veterinary Service, Eritrea; VRA: Veterinary Research Administration, Sudan; NK: not known; SR: streptomycin resistant mutant.

### Genome sequencing and identification of loci for MLSA

To identify the new loci for MLSA three near-complete genome sequences of differing Mccp strains (9231-Abomsa, 95043 and 97095-Tigray) were obtained by GATC Biotech AG (Konstanz, Germany). The genome sequence of strain 9231-Abomsa was obtained by 454 (Life Sciences, Roche, Basel, Switzerland) and verified by Solexa/Illumina (San Diego, USA) (correction of 40 nt residues). Sequences were assembled by GATC Biotech using Newbler, resulting in 50 large contigs, which were arbitrarily connected, providing a sequence of 1000 Kbp approximately. Lasergene SeqMan Pro V8 (DNAStar, Madison, USA) was used for all subsequent assemblies performed by the authors. The genome sequences of strains 95043 and 97095-Tigray were obtained by Solexa sequencing. They were assembled independently, using the 9231-Abomsa sequence as reference, and compared for the detection of polymorphic sites. Sequences showing insufficient cover (i.e.: less than five reads) as well as those exceeding twice the expected cover were excluded from this comparison. For the design of the MLSA system, several loci < 800 bp located within different contigs and showing multiple polymorphisms were selected. Both SNP and indels were considered, with the exception of indels in homopolymer sequences.

To identify the nature of the sequences corresponding to each locus, extended sequences including 2000 flanking nucleotides on either side of each locus were analysed using Vector NTI Advance™ 11.0 (Invitrogen Corporation, Carlsbad, USA). All identified open reading frames (ORF) were translated using the Mycoplasma/Spiroplasma genetic code and were compared by protein BLAST with the non redundant databases through the NCBI server [17].

### PCR and sequencing

Amplification of each of the locus sequences was performed in 50 μL reactions containing: 1 × *Taq *Buffer (Qiagen) with a final concentration of 1.5 mM MgCl_2_; 150 μM dCTP and dGTP; 300 μM dATP and dTTP; 0.4 μM each primer, 1 U *Taq *polymerase (Qiagen) and 1 ng of template. PCR reactions consisted in an initial denaturation step of 2 min at 94°C, followed by 35 cycles of 15 s at 94°C, 15 s at each corresponding annealing temperature and 30 s at 72°C. A final extension step was maintained for 5 min at 72°C. Primer sequences, annealing temperatures and PCR product sizes are shown in Table 2. The same primer pairs were used for sequencing of the corresponding PCR products by Beckman Coulter Genomics (Takeley, UK). The sequences obtained from each corresponding forward and reverse primer were assembled using Vector NTI Advance™11.0 (Invitrogen Corporation) and the extremities showing single strand sequences, as well as primer or aberrant sequences, were trimmed. All the corrected sequences obtained for each locus were aligned using ClustalW (Vector NTI) and were trimmed to the same size.

**Table 2:** Primer pairs developed in this study and variability of MLSA loci among 25 strains analysed

Locus	Primer name	Primer sequence (5'-3')	Annealing T° (°C)	Amplicon^a^ (bp)	Locus sequence b (bp)	Variable sites	Sequence types
Loc-01	MLSA-Mccp-01-F	GCTTATAGTGTTGTTGATACG	53	694	590	5	5
	MLSA-Mccp-01-R	GCAATAATCAATTAGCACAG					
Loc-03	MLSA-Mccp-03-F	ATTCCTCTCATTGAAGTTAC	47	765	664	7	7
	MLSA-Mccp-03-R	TAGATTAAGAGTCACAATGC					
Loc-11	MLSA-Mccp-11-F	TGATGGAATTATGTGTAGAGC	53	746	637	6	6
	MLSA-Mccp-11-R	ATGAACGATCTTGATGTTCC					
Loc-12	MLSA-Mccp-12-F	GGTATGGAGTTGATTTTGAAAC	58	742	646	4	4
	MLSA-Mccp-12-R	GCTCCAGCTAAAGCATTATTA					
Loc-15	MLSA-Mccp-15-F	GGACGAATTTATTTAGTTTCTGCTG	58	802	691	4	4
	MLSA-Mccp-15-R	ACATTAGTTTGCATACCACCAGTAA					
Loc-17	MLSA-Mccp-17-F	TAAACCAGAGCAAAACGGTA	58	751	649	8	6
	MLSA-Mccp-17-R	AACACTAACAATTCCAACAGC					
Loc-20	MLSA-Mccp-20-F	CTAGTTAATTTTGGAGCCGA	53	781	696	7	7
	MLSA-Mccp-20-R	CATCAATTGTTGATGAATCG					
H2^c^	N/A	N/A	N/A	N/A	2174	12	8
8 concatenated loci	N/A	N/A	N/A	N/A	6747	53	15

^a^Amplicon sizes in base pairs correspond to F38^T ^amplifications

^b^Locus sequence sizes in base pairs correspond to F38^T ^data

^c^For information regarding H2 locus amplification and sequencing refer to [8]

N/A: does not apply.

### Diversity analysis

The locus sequences corresponding to each strain were concatenated head-to-tail for diversity analyses conducted using Darwin 5.0 [18]. A distance tree was constructed using the neighbour-joining algorithm. Since our sample of strains was not a random representation of the Mccp population, the "unweighted" option was chosen. Because sequences were highly similar, the effect of multiple substitutions was considered negligible and no correction was applied to dissimilarities. The "pairwise gap block correction" option was selected with a minimal length for gap blocks of 1 nt. This implied that all consecutive gaps, starting from one nucleotide, were considered as a single event. Bootstrap analysis with 1000 replicates was performed.

## Results

### Choice of loci for MLSA

The H2 locus, which showed 12 polymorphic sites and had proven to be a valuable tool for Mccp typing [8], was retained for MLSA. The choice of additional molecular markers was done according to the analysis of polymorphisms observed among near-complete genome sequences of three Mccp strains corresponding to different H2 locus groups: 9231-Abomsa, 95043 and 97095-Tigray (Table 1). The aim was to mount up to around 50 polymorphic positions within seven or eight loci (standard for multi-locus sequence typing, MLST) to construct a discriminative typing tool.

Sequence comparisons between strain 9231-Abomsa and strains 95043 and 97095-Tigray respectively resulted in detection of over 1000 SNP within a genome of around 1000 Kbp. Twenty-two polymorphic loci were analysed using three additional strains corresponding to the most frequently represented H2 locus types: 94029-C5, 07033, and M74/93 (Table 1). This resulted in the selection of seven new variable loci (Table 2).

### Organisation of the MLSA loci

The sequences corresponding to the seven new loci were examined for functional categorisation by BLAST analysis. All except Loc-20 showed the highest similarity to sequences of the Mcc strain California kid (ATCC 27343) complete genome (CP000123). Most of the polymorphic sites were located in intergenic sequences and in what appeared to be pseudogenes, with the exception of 3 SNP in Loc-01 and 4 SNP in each Loc-12 and Loc-15, which were located in apparently full CoDing Sequences (CDS). Loc-01 was found to be homologous to the end of the 1-phosphofructokinase gene (*fruK*) of Mcc California kid (MCAP_0619) and to the intergenic sequence preceding MCAP_0620. Loc-03 corresponded to the end of a putative lipoprotein gene in Mcc California kid (MCAP_0782), which was truncated in Mccp, and to the beginning of a putative PTS system, IIBC component gene (MCAP_0783), also truncated in Mccp. Loc-11 showed greatest similarity to the ornithine carbamoyltransferase gene (*argF*) of California kid (MCAP_0654), which was truncated at both N- and C- terminal ends in Mccp. Loc-12 corresponded to the N-acetylglucosamine-6-phosphate deacetylase (*nagA*) of Mcc (MCAP_0438), whereas Loc-15 comprised the spermidine/putrescine ABC transporter permease component (*potB*, MCAP_0201) and permease and substrate-binding component (*potCD*, MCAP_0200). Loc-17 matched a putative membrane protein gene (MCAP_0137) truncated in Mccp. For Loc-20, no homologue was found in the California kid genome. The largest ORF in this locus showed 34% identity to the Maltodextrin ABC transporter permease gene (*malC*) of *Mycoplasma mobile *(MMOB3890), though the CDS was extensively truncated in Mccp. Finally, the organisation of the H2 locus has been previously described [8].

### Validation of the stability of the MLSA

The stability of the eight molecular markers selected for MLSA was assessed by analysis of epidemiologically related strains (isolated in nearby locations during an epizootic CCPP episode), as well as a variant obtained by in vitro passage. Two isolates originating from different locations but related to the same outbreak in Tunisia (Gabes and LKD, Table 1) and a subculture of strain Gabes after 102 in vitro passages were analysed for this purpose. The sequences corresponding to each of the eight loci of these three "variants" were identical, showing that the molecular markers were stable and there were no laboratory-introduced variations.

### Molecular typing and geographic distribution of Mccp strains

The MLSA strategy based on eight loci was extended to the strains listed in Table 1 with the exception of two strains used only as controls of the stability of the MLSA markers. The number of variables (SNP and indels) observed within each of the eight loci is indicated in Table 2. Fifteen different sequence types (ST) were discriminated among 25 strains, based on 53 polymorphisms.

The polymorphisms observed within each of the seven new loci among 25 strains are shown in Table 3. All the sequences were deposited in GenBank (Additional file 1, Table S1). As for the H2 locus, the sequences of eight strains that had not been previously analysed were determined in this study as previously described [8]. No original sequences were identified and corresponding H2 groups are shown in Table 1.

**Table 3:** Sequence polymorphisms among Mccp strains

	Loc-01					Loc-03							Loc-11						Loc-12				Loc-15				Loc-17								Loc-20						
**Position^a^**	**56**	**74**	**258**	**433**	**441**	**54**	**222**	**244**	**528**	**534-535**	**546**	**579**	**54**	**391**	**566**	**586**	**605-606**	**625**	**31**	**533**	**542**	**567**	**11**	**406**	**451**	**649**	**18**	**28**	**104**	**157-158**	**256**	**468**	**529**	**639**	**29**	**112**	**317**	**506**	**575**	**658**	**681**

F38^T^	G	A	C	A	A	C	G	C	A	-	T	G	C	G	C	G	-	C	A	A	G	G	T	C	T	T	G	C	C	-	A	A	G	T	T	A	G	C	T	C	T
97097-Errer	.	.	.	.	.	.	.	.	.	.	.	.	.	.	.	.	.	.	.	.	.	A	.	.	.	.	.	.	.	.	.	.	.	.	.	.	.	.	.	T	.
94029-C5	.	.	.	.	.	T	.	.	.	.	.	.	.	.	.	.	.	.	.	.	.	.	.	.	.	.	.	.	.	.	.	.	.	.	.	.	.	A	.	T	.
91039-C3	.	.	.	.	.	T	.	.	.	.	.	.	.	.	.	.	A	.	.	.	.	.	.	.	.	.	.	.	.	.	.	.	.	.	.	.	.	A	.	T	.
9231-Abomsa	.	.	.	.	.	T	.	.	.	.	.	.	.	.	.	.	A	T	.	.	.	.	.	.	.	.	.	.	.	.	.	.	.	.	.	.	.	A	.	T	.
Gabes	.	G	.	.	.	.	.	.	C	.	C	.	.	.	.	.	.	.	.	.	.	A	.	.	.	.	.	.	.	.	.	.	.	.	.	.	.	.	.	T	C
09018	.	G	.	.	.	.	.	.	C	.	C	.	.	.	.	.	.	.	.	.	A	A	G	.	.	.	.	.	.	.	G	.	.	G	.	.	.	.	C	T	C
7/2	.	G	.	.	.	.	.	.	C	.	C	.	.	.	.	A	.	.	.	.	.	A	.	.	.	.	.	.	.	.	.	.	.	.	.	.	.	.	.	T	C
M1601	.	G	.	.	.	.	.	T	C	.	C	.	.	.	.	.	.	.	.	.	A	A	G	.	.	.	.	T	.	.	G	.	.	G	.	.	.	.	C	T	C
C550/1	.	G	.	.	.	.	A	.	C	.	C	.	.	.	.	.	.	.	.	.	A	A	G	.	.	.	.	.	.	.	G	.	.	G	.	.	.	.	C	T	C
97095-Tigray	A	G	G	.	.	.	.	.	C	.	C	T	T	A	.	.	.	.	G	G	A	A	G	T	.	C	A	.	T	.	G	G	A	G	.	G	A	.	C	T	C
M74/93	A	G	G	.	G	.	.	.	C	.	C	T	T	A	.	.	.	.	G	G	A	A	G	T	.	C	A	.	T	.	G	G	A	G	.	G	A	.	C	T	C
95043	A	G	G	T	.	.	.	.	C	T	C	T	T	A	T	.	.	.	G	G	A	A	G	.	C	C	A	.	T	.	G	.	A	G	C	G	A	.	C	T	C
8789	A	G	G	T	.	.	.	.	C	T	C	T	T	A	T	.	.	.	G	G	A	A	G	.	C	C	A	.	T	T	G	.	A	G	.	G	A	.	C	T	C

^a^Position as on F38^T ^sequences.

**Figure 1:**
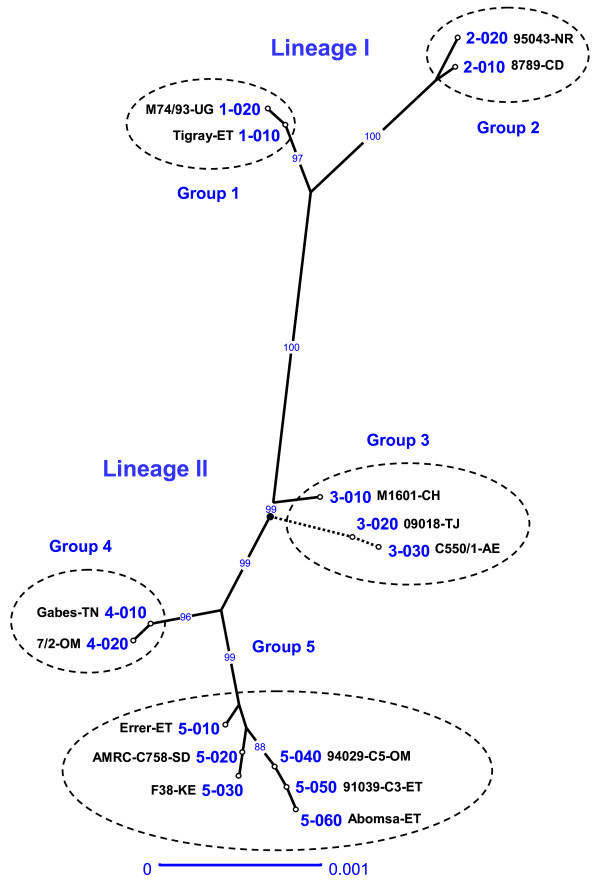
**Tree derived from distance analysis of the eight concatenated MLSA loci**. The tree was constructed using the unweighted neighbour-joining algorithm (Darwin 5.0). A single strain representing each of the 15 MLSA ST is displayed (see Table 1 for strain details). The two sequences presenting a large 960 nt deletion (09018 and C550/1) were grafted at their respective positions after tree construction (discontinuous lines) in order to avoid their influence during tree inference. The root of the tree is represented as a bold dot. Bootstrap percentage values were calculated from 1000 resamplings and values over 80% are displayed. The scale bar shows the equivalent distance to 1 substitution per 1000 nucleotide positions. The ST were numbered according to the group in which they clustered, followed by a three digit code that should allow intercalating additional ST as new sequences are obtained. Note that the two letter code following the strain name represents the corresponding country of isolation: AE, United Arab Emirates; CD, Chad; CH, China; ET, Ethiopia; NR, Niger; OM, Oman; SD, Sudan; TJ, Tajikistan; TN, Tunisia; UG, Uganda.

The polymorphisms found in each of the seven new MLSA loci for each ST are shown, represented by a single strain. Note that strain AMRC-C758 (representing MLSA type 5-020 in Figure 1) is identical to strain F38^T ^by analysis of the seven new loci shown here, though F38^T ^may be differentiated by H2 locus analysis (MLSA type 5-030). The GenBank accession numbers corresponding to F38^T ^locus sequences are as follows: Loc-01: HQ864744, Loc-03: HQ864761, Loc-11: HQ864776, Loc-12: HQ864786, Loc-15: HQ864807, Loc-17: HQ864814 and Loc-20: HQ864737. All accession numbers may be found in Additional file 1, Table S1.

**Figure 2:**
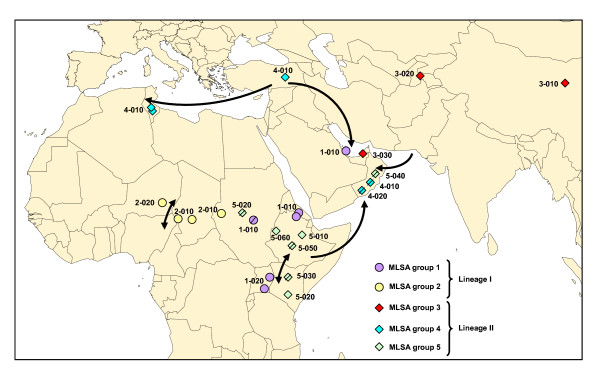
**Geographic origins of the strains analysed in this study**. Each strain is represented by a symbol corresponding to its MLSA group and by its ST. Note that when the exact location was not known the symbols were barred and were positioned arbitrarily in the corresponding country. The arrowed lines represent main routes of trade and other animal movements in the different areas.

The eight locus sequences corresponding to each strain were concatenated head-to-tail for sequence distance analysis. A robust tree (showing structured groups supported by high bootstrap values) was constructed using the neighbour-joining method (Figure 1). Two different lineages and five groups were identified. Lineage I was quite homogeneous and comprised two clusters: group 1, including strains from east Africa and an isolate from Qatar, and group 2, clustering strains from central Africa. Lineage II showed greater heterogeneity and comprised group 3, represented by strains from east Asia and an isolate from United Arab Emirates, group 4, including strains from north Africa, Turkey and the Arabian Peninsula, and group 5, represented by strains from east Africa and an isolate from Oman. A good correlation between MLSA groups and geographic origins was observed, with the exception of the Arabian Peninsula (Qatar, Oman, United Arab Emirates) wherein strains corresponding to four out of the five different groups could be found. Both groups 1 and 5, each corresponding to a different lineage, were present in east Africa. The geographic position of the different groups and sequence types is displayed in Figure 2.

Each of the five groups presented at least two different ST, allowing a further discrimination into subgroups. In group 1, the isolates from Uganda could be differentiated from all remaining strains. The Ethiopian and Eritrean strains were actually related since the goats responsible for the CCPP outbreak in Eritrea were imported from the Tigray province of Ethiopia, where the Ethiopian strain was isolated (Table 1). Group 2 could also be divided into two subgroups. This was the only clade that was not found in the Arabian Peninsula. Clade 3 was the most diverse, comprising three strains corresponding to three different ST. The Chinese isolate was the only one presenting a complete H2 locus, whereas strains from Tajikistan and the United Arab Emirates showed a previously described deletion of the H2 pseudogene [8]. Group 4 was rather homogeneous, comprising only two ST differentiated by a single SNP. One of the Omani isolates was actually of Turkish origin, corresponding to imported goats from Turkey (Table 1). Finally, group 5 was the best represented cluster, comprising eight strains corresponding to six different ST.

## Discussion

Sequence-based genotyping methods are technically simple, objective and portable [19]. The fact that these methods do not require isolation of the CCPP agent is particularly useful considering its fastidious nature. Direct amplification and sequencing from clinical material is possible even in cases where concomitant bacteria or antibiotic therapy hamper Mccp isolation, as was demonstrated in this work by analysis of a clinical sample from the Tajik outbreak. All these advantages had already been taken into consideration by the authors when developing an initial tool for Mccp typing based on the H2 locus [8]. The objective was now to evolve from a single locus sequence typing method to the analysis of multiple molecular markers in order to provide greater resolution by considerably increasing variability. Since the limited intraspecies variability of Mccp precluded the use of housekeeping genes, which constitute the standard molecular markers for MLST, the method was adapted to the analysis of other sequences, regardless of their coding capacity, which is currently known as MLSA. The selection of new molecular markers for MLSA was done in a comprehensive manner by comparison of near-complete genome sequences and identification of variable regions distributed along the Mccp genome. The resulting MLSA scheme based on eight loci revealed the presence of five distinct clades that were distributed in two evolutionary lineages.

This new MLSA scheme constitutes an important improvement from H2 locus typing, which was based on the analysis of a single genomic fragment of over 2000 bp [8]. MLSA resulted in a higher number of polymorphisms and an increased discriminatory power (53 polymorphisms providing 15 ST) compared to H2 locus sequence analysis (12 variables, 8 ST), as calculated by the comparison of 25 strains analysed (Table 2). Although some correlation was observed, H2 groups were not always consistent with MLSA groups. As an illustration, H2 locus group A clustered strains belonging to the two distinct MLSA lineages: Aa (corresponding basically to east African strains from lineage I) and A, Ab, Ac (mainly representing east African strains from lineage II). Though allowing a perfect correlation between cluster and geographic origin, H2 typing did not reveal the existence of two different lines of descent in east Africa. In conclusion, MLSA provided higher resolution for molecular typing whilst overcoming the bias of individual gene specificities, therefore better representing the evolution of Mccp strains.

Another study on the molecular evolution of Mccp had been previously performed based on 16S rDNA sequences [7,16]. Fifteen of the strains analysed by 16S rDNA were also analysed in this work, allowing a direct comparison of the results (Table 1). First, the two lineages evidenced by MLSA analysis correlated well with those obtained using 16S rDNA sequences, supporting the evolutionary significance of these two major lines of descent, which were also supported by the analysis of several housekeeping genes [6]. Moreover, the MLSA strategy provided similar resolution than that obtained by 16S rDNA analysis, though based on a much higher number of polymorphisms. When taking into consideration the 15 strains common to both studies, 12 ST were discriminated by 16S rDNA analysis according to 16 polymorphisms, whereas 10 ST were obtained by MLSA based on 49 polymorphisms. However, this must be regarded with care as two of the ST discriminated by 16S rDNA typing were directly related to the presence of a single mutation conferring streptomycin resistance. Since the appearance of this mutation was not related to the natural evolution of the strains baring it but to the selective pressure resulting from exposure to streptomycin, either in the field or in the laboratory, the corresponding SNP should not be retained for molecular epidemiology analysis. This would reduce to 10 ST the number of evolutionary significant types obtained by 16S rDNA. In conclusion, MLSA provided the same resolution as 16S rDNA analysis for molecular typing of Mccp strains, while allowing the discrimination of five evolutionary groups consistent with CCPP epidemiological data.

A discriminative tool for the molecular typing of Mccp strains has been developed here. The main limitation to study the molecular epidemiology of CCPP remains the lack of Mccp strains or DNA samples for analysis. However, the analysis of genetic data generated in this work has provided some answers to the initially posed questions.

A good correlation between MLSA groups and geographic origins of the strains was observed. The only exception to this was the Arabian Peninsula, wherein Mccp strains corresponding to several evolutionary groups were found. This may be explained by the frequent importation of animals from diverse origins, particularly for the Muslim feasts celebrated every year. Otherwise, the geographic distribution of the MLSA groups was quite explicit.

A distinct Asian clade was identified by MLSA, represented by two strains from Tajikistan and China and also comprising a strain from Dubai. In spite of the sampling limitation, the existence of this clade strongly suggests that these strains have evolved locally and, therefore, that they have not been introduced recently in this continent. CCPP was suspected long ago in continental Asia based on historical clinical descriptions, with substantiating data presented in India already in 1914 [20]. The recent declaration of the disease in Tajikistan should encourage neighbouring countries to search for Mccp, enabling a better assessment of the distribution of CCPP in Asia.

A local evolution of strains was also demonstrated in central Africa, where a single MLSA group was observed. Furthermore, this group was restricted to central Africa, constituting the only clade that was not identified in the Arabian Peninsula. Although, arguably, this could be attributed to insufficient sampling, the limited animal movements in this region, where transhumance is oriented north-south, may well explain the exclusive presence of indigenous strains. This leads us to reject the assumption that CCPP was introduced from east Africa, as it was proposed in 1987 when the disease was first discovered in Chad [21]. Also, if we consider the distribution of other contagious diseases of goats such as "peste des petits ruminants", we may suspect that CCPP is also present in west Africa. An active search for the etiologic agent should be encouraged to elucidate the western limits of the distribution of CCPP in Africa.

Isolates from north Africa and Turkey corresponded to the same MLSA group, which reflects the importance of Mediterranean trading routes, particularly the exportation of animals from Turkey to north Africa and the Arabian Peninsula. CCPP has been known for many years in Turkey, where it appears to be widespread. Moreover, uncontrolled animal movements in this region should raise suspicions regarding the presence of the disease in neighbouring countries [22]. Further strains should be typed to assess the variability existing within Turkey, while efforts should be made at a regional level to better understand the distribution of the disease in this area. This applies also to north Africa, where the presence of the disease was confirmed in 1980 in Tunisia [23], though no further studies have been published since then.

In east Africa two MLSA groups, each belonging to a different lineage, were identified. Strains belonging to each of the two evolutionary lines have been spreading in this region over the last decades and the disease has recently reached the Indian Ocean [10].

**Figure 3:**
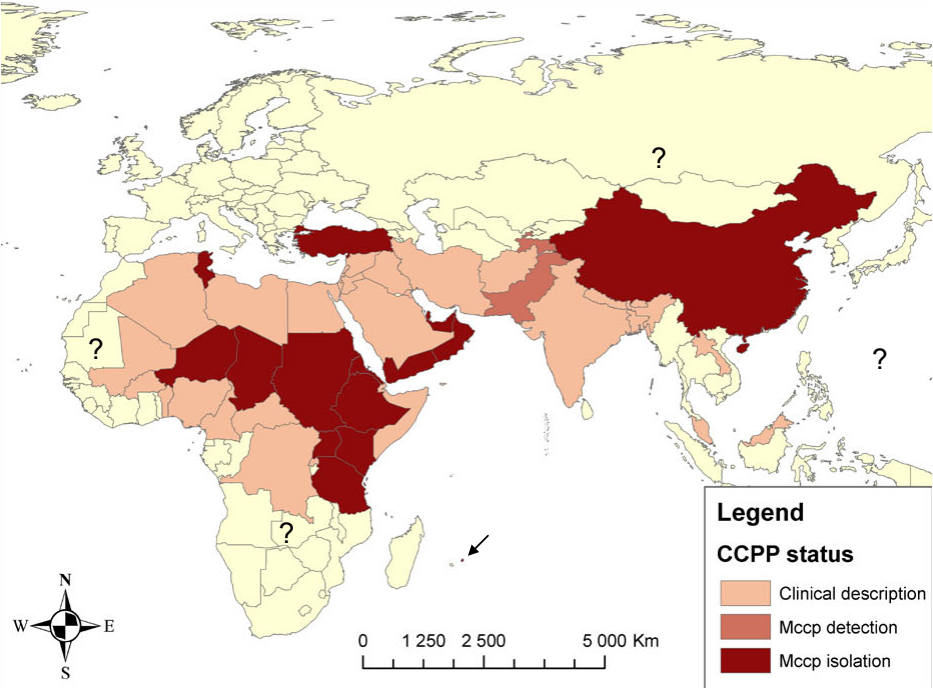
**Probable distribution of CCPP**. The countries in which the disease has been described, those in which the etiologic agent has been detected using molecular tests and those in which it has been isolated are indicated. The arrow indicates the presence of the disease in Mauritius, where Mccp was isolated in 2009.

Although the recent confirmation of the presence of CCPP in continental Asia has provided a better estimation of the distribution of CCPP world-wide, some questions still remain. CCPP has only been reported in sixteen countries, while, if we take into consideration reports of clinical disease, over forty countries of Africa and Asia may be affected. The boundaries of the disease in Asia, as well as towards the west and south of the African continent are still uncertain but, taking into consideration the contagiousness of the disease and the movements of nomadic goat herds, CCPP is probably present in central and north-east Africa, the Middle East and all the way through to China. Figure 3 shows an updated map presenting the probable distribution of CCPP.

Mccp has been isolated from sheep showing clinical disease that had been in contact with CCPP-infected goats [22,24] and the existence of the disease in wildlife species was also demonstrated recently in Qatar [14]. Although the origin of this outbreak was not elucidated, it was proposed that the wild species kept in a conservation area may have been contaminated by domestic goats. Actually, two of the strains here analysed corresponded to these reports: a sheep isolate from Uganda [24] and another one originating from a wild goat in Qatar [14]. Both of them shared ST with other goat isolates, suggesting that the same strains can affect a wide range of species. However, further studies are required to identify the genetic determinants of species-specificity. Also, the role of these species in the epidemiology of CCPP is yet to be elucidated.

A discriminative MLSA scheme has been designed as a tool for the molecular epidemiology of CCPP. It would be advantageous to form a publicly accessible database that will be enriched by sequences obtained by different laboratories in affected countries. Such a database could be linked to the websites of the OIE [25] and FAO [26], which provide updated information regarding new Mccp outbreaks. This new typing tool may help improve the surveillance and control of the disease, as well as to trace new epidemics.

## Competing interests

The authors declare that they have no competing interests.

## Authors' contributions

FT and LMS conceived the study and participated in its design and coordination. YC provided material and data from Chinese isolates. VD and LMS carried out the analysis. LMS drafted the manuscript, with the collaboration of FT, VD and YC. All authors read and approved the final manuscript.

## Supplementary Material

Additional file 1 **Table S1. GenBank accession numbers of locus sequences obtained in this study**. Displayed are GenBank accession numbers corresponding to the sequences of 14 strains representing the 14 ST discriminated based on the seven new MLSA loci. Click here for file
