# Supplementary material for: Multi-locus sequence analysis of mycoplasma capricolum subsp. capripneumoniae for the molecular epidemiology of contagious caprine pleuropneumonia
Source: Vet Res. 2011 Jul 14;42(1):86. doi: 10.1186/1297-9716-42-86 (PMC3177781; doi:10.1186/1297-9716-42-86)
Supplement: Additional file 1 — Table S1. GenBank accession numbers of locus sequences obtained in this study. Displayed are GenBank accession numbers corresponding to the sequences of 14 strains representing the 14 ST discriminated based on the seven new MLSA loci. [file 1297-9716-42-86-S1.DOC]

**Table S1**: GenBank Accession numbers of locus sequences obtained in this study

| **Strain** | **Loc-01** | **Loc-03** | **Loc-11** | **Loc-12** | **Loc-15** | **Loc-17** | **Loc-20** |
| --- | --- | --- | --- | --- | --- | --- | --- |
| F38^T^ | HQ864744 | HQ864761 | HQ864776 | HQ864786 | HQ864807 | HQ864814 | HQ864737 |
| 97097-Errer | HQ864748 | HQ864762 | HQ864774 | HQ864791 | HQ864813 | HQ864820 | HQ864738 |
| 94029-C5 | HQ864747 | HQ864760 | HQ864782 | HQ864789 | HQ864812 | HQ864819 | HQ864742 |
| 91039-C3 | HQ864745 | HQ864758 | HQ864780 | HQ864787 | HQ864810 | HQ864817 | HQ864743 |
| 9231-Abomsa | HQ864746 | HQ864759 | HQ864781 | HQ864788 | HQ864811 | HQ864818 | HQ864741 |
| Gabes | HQ864749 | HQ864763 | HQ864777 | HQ864790 | HQ864808 | HQ864815 | HQ864739 |
| 09018 | HQ864756 | HQ864766 | HQ864784 | HQ864794 | HQ864805 | HQ864822 | HQ864734 |
| 7/2 | HQ864754 | HQ864764 | HQ864778 | HQ864792 | HQ864809 | HQ864816 | HQ864740 |
| M1601 | HQ864757 | HQ864767 | HQ864785 | HQ864795 | HQ864806 | HQ864823 | HQ864736 |
| C550/1 | HQ864755 | HQ864765 | HQ864779 | HQ864793 | HQ864804 | HQ864821 | HQ864735 |
| 97095-Tigray | HQ864752 | HQ864768 | HQ864783 | HQ864797 | HQ864800 | HQ864825 | HQ864733 |
| M74/93 | HQ864753 | HQ864769 | HQ864775 | HQ864799 | HQ864801 | HQ864826 | HQ864732 |
| 95043 | HQ864751 | HQ864771 | HQ864773 | HQ864796 | HQ864803 | HQ864824 | HQ864730 |
| 8789 | HQ864750 | HQ864770 | HQ864772 | HQ864798 | HQ864802 | HQ864827 | HQ864731 |

Only the sequences of 14 strains representing the 14 ST discriminated based on the seven new MLSA loci have been deposited. The corresponding sequences of strains F38^T^ (5-030) and AMRC-C758 (5-020) are identical, though these strains may be differentiated by H2 locus analysis.
